# News and Notes

**Published:** 1898-08

**Authors:** 


### *NEWS AND NOTES.*

---

DRS. W. S. ELKIN, C. E. Murphy and F. W. McRae attended the last annual meeting of the American Medical Association, in Denver, Col. They report a most delightful trip and a most prosperous meeting.

---

DR. G. P. ROBINSON, late of Boston, Mass., has settled in Atlanta. Dr. Robinson will limit himself to the practice of diseases of children. We present in this issue an article from his pen on "Infant Feeding."

---

DR. F. W. MCRAE, of this city, was recently elected to deliver the annual address on surgery at the next meeting of the American Medical Association, in Columbus, Ohio. This is a compliment worthily bestowed. Medical journals will please note that the name is McRae and not McCrea.

---

DR. FRANK S. BOURNS, of this city, has been appointed chief surgeon on General Merritt's staff, and perhaps has already arrived at the Philippine Islands. Dr. Bourns at one time lived for four years upon the Islands, and this previous knowledge will be of great value to General Merritt as well as to the doctor himself in the discharge of his new duties. Dr. Bourns expects to return to Atlanta and be connected with the new College of Physicians and Surgeons.

---

DR. LUTHER B. GRANDY, Atlanta, Ga., has been appointed surgeon, with the rank of major, of the Third Regiment Georgia Volunteers. Governor Atkinson has before this distinguished himself by the excellence of his medical appointments. Scarcely could a more popular Southern surgical commission have been issued than the one to Dr. Grandy. He leaves a large and influential practice in Atlanta to respond to the call of his country.— *Va. Med. Semi-Monthly.*

---

FORT MCPHERSON, one of the army posts erected in recent years, is one of the best in the South. It is situated just outside of Atlanta's limit and readily accessible by street-car lines. Since the beginning of the Spanish-American war there has been opened a large general hospital, to which the wounded may be sent. It will perhaps be the central point for the South, in that Atlanta is so accessible. The hospital can accommodate four hundred patients, and perhaps more if it was found necessary. The main buildings are four in number and two stories in height. Each building is about 100 feet in length, has porches in the front and back, and has four main wards on the upper floor, with a number of smaller wards, wash-rooms, kitchens, etc., on the lower floor. The wards are well lighted and well ventilated. There is, in addition, the regular post hospital reserved for officers. Each ward is used for the reception of one class of cases. At present there are occupied two surgical wards, one fever ward, one ward for general medical cases and convalescents, and one for venereal cases. The nursing is in the hands of a trained hospital corps, and this will soon be supplanted by a force of trained female nurses.

---

DR. S. C. AYERS, of Cincinnati, has been elected professor of ophthalmology in the Medical College of Ohio, the medical department of the University of Cincinnati.

THESE drug houses notify us that they will stand the revenue-tax on their preparations: Peacock Chemical Co. and Sultan Drug Co., St. Louis.

---

JEFFERSON Medical College, of Philadelphia, is preparing to erect a new college building on the old site. It will be eight stories high, and when completed will be most perfect in every detail.

---

COMMODORE GEORGE DEWEY, the hero of Manila, is the son of Dr. Julius Y. Dewey, first president and medical director of the National Life Insurance Co. of Vermont. His brothers still live there.

---

PROFESSOR ADAM POLITZER, the well-known otologist of Vienna, will soon occupy his own chair and that of Professor Gruber in the University of Vienna, since the latter has resigned this chair, which he has held so long and so faithfully.

---

WE note that some of the most distinguished American surgeons have given up a lucrative practice in order to offer their services to their country. Some of those who have enlisted are Drs. Nicolas Senn, George Ryerson Fowler, Wm. H. Daly, Donald McLean, and a host of other well-known surgeons.

---

MEMPHIS has just completed its new City Hospital, at a cost of $70,000. It is first-class in every respect, being supplied with enameled bedsteads, electric elevators, indoor telephones, a handsome and thoroughly sterilizable operating-room, etc. There will be a training-school for nurses attached to the hospital.

---

THE American Association of Genito-Urinary Surgeons met at West Point June 7th and 8th. Dr. James Bell, of Montreal, was made president, Dr. Samuel Alexander, of New York, vice-president, and Dr. William K. Otis, of New York, secretary and treasurer. The next meeting will be at Niagara Falls, May, 1899.

---

THE Memphis Hospital Medical College has decided for the future to postpone the regular commencement of the work of the curriculum until November 1st, for the reason that this date better suits the convenience of the students, whose tuition-fees are only forthcoming after the harvesting of the cotton crop. This arrangement will prove beneficial to both sides.

---

IN view of persistent charges of mismanagement preferred by Dr. Chas. B. Kelsey, who was formerly a member of the faculty of the New York Post-Graduate School, the State Board of Charities has finally decided to investigate the matter, and the same is now going on behind closed doors. The chief charge seems to be that money donated for the endowment of special beds or other purposes had been used for paying current expenses, a total of $85,000 having been thus diverted.

---

MR. DAVID H. KING, JR., of New York, has offered to the government the use of his residence and estate on Jekyl Island, off Brunswick, Ga., without cost or restriction, as a military hospital until the close of the war. Jekyl Island is owned by a club, and is rarely visited by any but its members. Half a million dollars have been expended upon it, and it has been transformed from dreary sand and rioting foliage into a beautiful pleasure ground. The residence is a new and handsome one and would form an ideal convalescent hospital for wounded soldiers and sailors.—*Med. News.*

---

THE Marine Hospital Service, July 9, gave out the following copy of a report from the surgeon in charge at McHenry, Miss., announcing the discharge of the last case of yellow fever at that point. It was dated July 8, and reads: "Last of yellow fever discharged; tent and bedding in steam disinfector; under treatment, none; suspicious, none; will continue doing sanitary work." This leaves the entire country without a case ef yellow fever. While the cases at McHenry have been under treatment surgeons of the Marine Hospital Service have been inspecting and watching carefully many other points where it was thought possible the fever might appear, but no cases have developed. The success which has followed the confining and stamping out of the disease at McHenry is attributed to the early knowledge of the first case and the rigorous and painstaking action taken immediately by the Service and State authorities. The State authorities have cooperated in the work of watching for and controlling the disease. The Service has kept under surveillance not only the sick and suspicious, but has followed refugees to many points and kept watch over them as a safeguard against any possibility of the disease appearing in any quarter and remaining temporarily undiscovered. The statement given by the Marine Hospital Service makes the total number of cases at McHenry 23; at Euculla, 1; total, 24; deaths, none.

---

THE FIRST NATHAN LEWIS HATFIELD PRIZE FOR ORIGINAL RESEARCH IN MEDICINE.—The College of Physicians of Philadelphia announces through its committee that the sum of five hundred dollars will be awarded to the author of the best essay in competition for the above prize. Subject: "A Pathological and Clinical Study of the Thymus Gland and its Relations."

Essays must be submitted on or before January 1st, 1900. Each essay must be typewritten, designated by a motto or device, and accompanied by a sealed envelop bearing the same motto or device and containing the name and address of the author. No envelope will be opened except that which accompanies the successful essay.

The committee will return the unsuccessful essays if reclaimed by their respective writers or their agents within one year. The committee reserve the right not to make an award if no essay submitted is considered worthy of the prize.

The treatment of the subject must, in accordance with the conditions of the Trust, embody original observations or researches or original deductions. The competition shall be open to members of the medical profession and men of science in the United States.

The original of the successful essay shall become the property of the College of Physicians. The trustees shall have full control of the publication of the memorial essay. It shall be published in the Transactions of the College, and also, when expedient, as a separate issue. Address J. C. Wilson, Chairman College Physicians, 219 South Thirteenth St., Philadelphia, Pa.

---

MRS. FADDE, Faith-curist: " How is your grandfather this morning, Bridget?"

Bridget: "He still thinks he has the rheumatism mighty bad, mum."

"You mean he thinks he has the rheumatism. There is no such thing as rheumatism."

A few days later:

"And does your grandfather still persist in his delusion that he has rheumatism?"

"No, mum; the poor man thinks now thot he is dead. We buried um yisterday."—*Indianapolis Journal.*

---

*Cassidy* (reading)—It siz here that boicoicle scorching makes a mon gray-haired, round-shouldered, narrow-chisted, bow-legged, saller-faced and hump-backed.

*Regan* (who is deaf)—Behivins! that's only too true, Cassidy; and still min will kape on gittin' married.—*Puck.*

